# Effect of the Glucagon-Like Peptide-1 Receptor Agonists Semaglutide and Liraglutide on Kidney Outcomes in Patients With Type 2 Diabetes: Pooled Analysis of SUSTAIN 6 and LEADER

**DOI:** 10.1161/CIRCULATIONAHA.121.055459

**Published:** 2021-12-14

**Authors:** Ahmed M. Shaman, Stephen C. Bain, George L. Bakris, John B. Buse, Thomas Idorn, Kenneth W. Mahaffey, Johannes F.E. Mann, Michael A. Nauck, Søren Rasmussen, Peter Rossing, Benjamin Wolthers, Bernard Zinman, Vlado Perkovic

**Affiliations:** Sydney School of Public Health, University of Sydney, Australia (A.M.S.).; The George Institute for Global Health, The University of New South Wales, Sydney, Australia (A.M.S., V.P.).; College of Pharmacy, King Saud University, Riyadh, Saudi Arabia (A.M.S.).; Institute of Life Science, Swansea University Medical School, Singleton Hospital, Swansea, United Kingdom (S.C.B.).; University of Chicago Medicine, IL (G.L.B.).; University of North Carolina School of Medicine, Chapel Hill (J.B.B.).; Novo Nordisk A/S, Søborg, Denmark (T.I., S.R., B.W.).; Stanford Center for Clinical Research (SCCR), Department of Medicine, Stanford School of Medicine, CA (K.W.M.).; KfH Kidney Center, Munich, Germany (J.F.E.M.).; Friedrich Alexander University, Erlangen, Germany (J.F.E.M.).; Diabetes Division, Katholisches Klinikum Bochum, St Josef Hospital (Ruhr-Universität Bochum), Bochum, Germany (M.A.N.).; Steno Diabetes Center Copenhagen, Gentofte, Denmark (P.R.).; Department of Clinical Medicine, University of Copenhagen, Denmark (P.R.).; Lunenfeld–Tanenbaum Research Institute, Mt Sinai Hospital, University of Toronto, Canada (B.Z.).

**Keywords:** albuminuria, chronic kidney disease, eGFR, glucagon-like peptide-1 receptor agonists, liraglutide, semaglutide, type 2 diabetes

## Abstract

We assessed the effect of once-weekly semaglutide and once-daily liraglutide on kidney outcomes in type 2 diabetes.

**Methods:**

Pooled (n=12 637) and by-trial data from SUSTAIN 6 (Trial to Evaluate Cardiovascular and Other Long-Term Outcomes With Semaglutide in Subjects With Type 2 Diabetes; n=3297) and LEADER (Liraglutide Effect and Action in Diabetes: Evaluation of Cardiovascular Outcome Results; n=9340) were assessed for albuminuria change, annual slope of estimated glomerular filtration rate (eGFR) change, and time to persistent eGFR reduction (30%, 40%, 50%, and 57%) from baseline.

**Results:**

The median follow-up durations were 2.1 years for SUSTAIN 6 and 3.8 years for LEADER. In the pooled analysis, semaglutide/liraglutide lowered albuminuria from baseline to 2 years after randomization by 24% versus placebo (95% CI, 20%–27%; *P*<0.001). Significant reductions were also observed in by-trial data analyses (*P*<0.001 for all), the largest being with semaglutide 1.0 mg (33% [95% CI, 24%–40%]; *P*<0.001) at 2 years. With semaglutide 1.0 mg and liraglutide, eGFR slope decline was significantly slowed by 0.87 and 0.26 mL/min/1.73 m^2^/y (*P*<0.0001 and *P*<0.001), respectively, versus placebo. Effects appeared larger in patients with baseline eGFR <60 versus ≥60 mL/min/1.73 m^2^ (*P*_interaction_=0.06 and 0.008 for semaglutide 1.0 mg and liraglutide, respectively). Semaglutide/liraglutide significantly lowered risk of persistent 40% and 50% eGFR reductions versus placebo (hazard ratio [HR], 0.86 [95% CI, 0.75–0.99]; *P*=0.039 and HR, 0.80 [95% CI, 0.66–0.97]; *P*=0.023, respectively). Similar, nonsignificant, directional results were observed for 30% and 57% eGFR reductions (HR, 0.92 [95% CI, 0.84–1.02]; *P*=0.10 and HR, 0.89 [95% CI, 0.69–1.13]; *P*=0.34). In patients with baseline eGFR 30 to <60 mL/min/1.73 m^2^, the likelihood of persistent reduction for all thresholds was increased, ranging from HR 0.71 for 30% reduction (95% CI, 0.59–0.85; *P*=0.0003, *P*_interaction_=0.017) to 0.54 for 57% reduction (95% CI, 0.36–0.81; *P*=0.003, *P*_interaction_=0.035).

**Conclusions:**

In patients with type 2 diabetes, semaglutide/liraglutide offered kidney-protective effects, which appeared more pronounced in patients with preexisting chronic kidney disease.

## Abstract 2

Supplemental Digital Content is available in the text.

**Clinical Perspective**

**What Is New?**

- Data suggest a kidney-protective effect with glucagon-like peptide-1 receptor agonists driven primarily by beneficial albuminuria outcomes, but definitive data on more severe kidney outcomes are lacking.
- In this analysis of SUSTAIN 6 (Trial to Evaluate Cardiovascular and Other Long-Term Outcomes With Semaglutide in Subjects With Type 2 Diabetes) and LEADER (Liraglutide Effect and Action in Diabetes: Evaluation of Cardiovascular Outcome Results), we demonstrate the benefit of using once-weekly semaglutide and once-daily liraglutide on a number of clinically important kidney outcomes: changes in albuminuria, annual slope of estimated glomerular filtration rate change, time to persistent proportional estimated glomerular filtration rate reductions of 40% and 50% from baseline, and a composite end point (time from randomization to first occurrence of kidney failure/death or proportional estimated glomerular filtration rate decline).

**What Are the Clinical Implications?**

- The results of this study suggest that the glucagon-like peptide-1 receptor agonists semaglutide and liraglutide may add to the kidney-protective treatment options available to people with type 2 diabetes and diabetic kidney disease.
- Further studies are required to investigate the full effect of glucagon-like peptide-1 receptor agonists on primary kidney end points in dedicated trials in diabetic kidney disease. SUSTAIN 6 and LEADER examined kidney end points as secondary outcomes and enrolled a majority of patients without diabetic kidney disease but with cardiovascular disease.

Type 2 diabetes (T2D) is a major risk factor for the development and progression of chronic kidney disease, commonly referred to as diabetic kidney disease (DKD).^1^ Approximately 50% of people with T2D will develop DKD in their lifetime, and approximately half of kidney failure cases are ascribed to diabetes.^1,2^ As the global burden of T2D is increasing,^3^ so is the prevalence of kidney failure. The prevalence of kidney failure is predicted to exceed 5 million people by 2030.^4^

People with DKD have a high risk of cardiovascular morbidity and mortality and generate increased costs associated with their treatment.^5^ A limited number of treatments have been shown to lower this risk. Therefore, it is important to identify treatments that reduce the risk of cardiovascular complications and of progression to kidney failure in people with DKD.

Recent developments in the management of T2D have identified treatments that reduce the risk of DKD progression. In cardiovascular outcome trials, sodium-glucose cotransporter-2 inhibitors and glucagon-like peptide-1 receptor agonists (GLP-1 RAs) have lowered the risk of major adverse cardiovascular events.^6–12^ Secondary kidney outcomes in these cardiovascular outcome trials have suggested kidney benefits.^6–16^ In the first designated T2D kidney outcome trials, the sodium-glucose cotransporter-2 inhibitors canagliflozin and dapagliflozin were shown to substantially lower the risk of hard kidney outcomes compared with placebo (patients with and without T2D were included in the latter trial).^17,18^

This post hoc analysis aimed to evaluate the effect of once-weekly semaglutide and once-daily liraglutide therapy on a broad range of clinically important kidney outcomes, including changes in albuminuria, estimated glomerular filtration rate (eGFR) slope, and persistent reductions in eGFR.

## Methods

### Data Sharing Statement

Deidentified individual participant data, the study protocol, and a redacted clinical study report will be available according to Novo Nordisk data sharing commitments. The data will be made available permanently after research completion and approval of product and product use in both the European Union and United States. Data will be shared with bona fide researchers submitting a research proposal requesting access to data and for use as approved by the independent review board according to the independent review board charter. This and the access request proposal form and access criteria can be found at novonordisk-trials.com. The data will be made available on a specialized SAS data platform.

### Trial Designs and Patients

SUSTAIN 6 (Trial to Evaluate Cardiovascular and Other Long-Term Outcomes With Semaglutide in Subjects With Type 2 Diabetes; URL: https://www.clinicaltrials.gov; Unique identifier: NCT01720446) and LEADER (Liraglutide Effect and Action in Diabetes: Evaluation of Cardiovascular Outcome Results; URL: https://www.clinicaltrials.gov; Unique identifier: NCT01179048) were randomized, double-blinded, placebo-controlled trials. Detailed methods of both trials have been published previously.^6,7^ The 2 trials included patients with T2D at high risk of cardiovascular events and with a glycohemoglobin (HbA1c) level ≥7%. In SUSTAIN 6, patients were randomized to once-weekly semaglutide 0.5 mg subcutaneously, once-weekly semaglutide 1.0 mg subcutaneously, or matching placebo for 2 years. In LEADER, patients were randomized to once-daily liraglutide up to 1.8 mg subcutaneously or matching placebo for 3.5 to 5 years (median 3.8 years). The primary outcome in both trials was major adverse cardiovascular events, consisting of nonfatal myocardial infarction, nonfatal stroke, or cardiovascular death. Kidney end points were collected as secondary outcomes. Both cardiovascular and kidney outcomes were adjudicated by an external blinded event adjudication committee.^6,7^

In SUSTAIN 6, serum creatinine was collected at screening, randomization (baseline), and after baseline at weeks 2, 4, 8, 16, 30, 44, 56, 68, 80, 92, and 104. Calculation of eGFR was performed using the MDRD equation (modification of diet in renal disease).^19^ Urinary albumin-to-creatinine ratio (UACR) was collected at baseline and after baseline at weeks 16, 30, 44, 56, 80, and 104.

In LEADER, serum creatinine was collected at screening, randomization (baseline), and after baseline at the 6-month visit, and then annually until final visit. Calculation of eGFR was performed using the MDRD equation.^19^ UACR was collected at baseline and after baseline annually until final visit.

Urinary albumin values below the lower limit of quantification of 3 mg/g were imputed as 1.5 mg/g (lower limit of quantification/2) in the post hoc calculation of UACR. Approximately 17% of randomized patients had UACR values below the lower limit of quantification in both SUSTAIN 6 and LEADER at baseline.

### Subgroups

We evaluated outcomes for the overall pooled population and according to preexisting kidney disease, defined by baseline eGFR (using the MDRD formula) and albuminuria. Patients were stratified by baseline eGFR (≥90 mL/min/1.73 m^2^, 60–<90 mL/min/1.73 m^2^, 30–<60 mL/min/1.73 m^2^, or <30 mL/min/1.73 m^2^) and albuminuria stage (normoalbuminuria [UACR <30 mg/g], microalbuminuria [UACR 30−300 mg/g], and macroalbuminuria [UACR >300 mg/g]). Effects of semaglutide and liraglutide versus placebo on albuminuria over time, average annual eGFR decline (slope), and eGFR at the 2-year visit were also evaluated in the populations treated with semaglutide 0.5 and 1.0 mg and liraglutide 1.8 mg, respectively.

### Outcomes

We explored the effects of semaglutide in SUSTAIN 6 and liraglutide in LEADER, compared with placebo, on clinically important kidney outcomes,^20^ including change in albuminuria from baseline to the 2-year visit (defined as UACR estimated ratio to baseline and presented as estimated geometric mean ratio between treatment and placebo), annual change in eGFR from baseline (total slope), and time to persistent 30%, 40%, 50%, and 57% eGFR (57% being equivalent to doubling of creatinine) reductions from baseline, defined by time to first occurrence from randomization of the relevant reduction confirmed by a subsequent measurement. If no subsequent visit was performed, the confirmation was omitted. The CKD-EPI equation (Chronic Kidney Disease Epidemiology Collaboration) was used to calculate the eGFR used in the total slope analysis.

Other outcomes included a composite of time from randomization to first occurrence of kidney death, need for maintenance kidney replacement therapy, or first occurrence of a reduction in eGFR of 40%, 50%, or 57% (each percentage analyzed separately with the first 2 components, to give 3 composite end points). We estimated total loss of eGFR over 2 years from randomization for both trials according to treatment groups. We assessed these outcomes in the overall pooled population and in subgroups defined by UACR and eGFR at baseline, as defined previously. Kidney failure and death, as components of the original secondary end points of both trials, were confirmed by the event adjudication committee.^6,7^

### Statistical Analysis

Data from all randomized patients in LEADER and SUSTAIN 6 were included from date of randomization to the end of follow-up visit. In the pooled analyses, both semaglutide treatment arms and the liraglutide treatment arm were pooled and compared with placebo. For the pooled analyses, the trial was used as a fixed effect.

Effects of semaglutide and liraglutide by trial on albuminuria over time as compared with placebo were assessed using a mixed model for repeated measures with UACR as dependent variable (which was log-transformed owing to the nonnormal distribution), treatment and visits as fixed factors, baseline UACR (log-transformed) as a covariate, and the interactions between visits and treatment/baseline UACR, respectively. An unstructured covariance matrix for repeated measures was used. Least square means, differences, and 95% CIs between treatments were then back-transformed to the original scale. Because of the different trial durations of LEADER and SUSTAIN 6, treatment ratios were evaluated at 2 years from randomization to align comparisons of both trials. Pooled analyses were done using the same model with trial included as an additional factor.

Slope analyses of eGFR were performed using a random-slope model by trial, with change from baseline as dependent variable, baseline value and time (in years) as covariate, treatment as a fixed factor, and the interaction between treatment and time. The intent-to-treat populations were used. Changes from baseline were assessed between each visit and used as the repeated measure, with the time from randomization as the underlying continuous time scale for the slope analyses. Patient-specific intercepts and time as random effects assuming a bivariate normal distribution for these effects were included in the model. Analyses by subgroups were performed by including the respective subgroups as a fixed factor and the interaction with treatment.

Time-to-first-event analyses were performed using Cox proportional hazard models, with pooled treatment as a fixed factor and stratified by trial. Patients without respective events were censored at death or end of follow-up, whichever came first. Time to persistent reduction of eGFR from baseline (30%, 40%, 50%, and 57%) was analyzed independently from each other. Subgroup analyses were performed by including subgroup as a fixed factor and the interaction between subgroup and treatment. A quadratic spline Cox regression model was also used to analyze the time to persistent reduction end points (30%, 40%, 50%, 57%), showing the hazard ratios (HRs) between the treatments (pooled GLP-1 RAs vs placebo) according to continuous measures.

An *I*^2^ test was used to measure the heterogeneity between the 2 trials when assessing the effect of semaglutide and liraglutide versus placebo on the annual eGFR slope at 2 years visit in the overall population and subgroups with preexisting DKD defined by the level of albuminuria and eGFR at baseline.

No adjustment for multiplicity or missing values was performed. A significance level of 5% was used overall. Statistical analysis was performed with SAS version 9.4 (SAS Institute).

### Ethics

Both studies received full approval by ethics committees or institutional review boards at each participating site (names and locations of sites are available in the supplementary information of the primary publications of both trials^6,7^) and were conducted according to the principles of the Declaration of Helsinki.^21^ All participants provided written informed consent before participation in trial-related activities.^6,7^

### Role of the Funding Source

The sponsor participated in the study design and management, analysis, and interpretation of data. Three of the authors of this article are employees of the sponsor and, as such, were involved in the preparation, review, and approval of the article. All authors had full access to all the data in the study and had final responsibility for the decision to submit for publication.

## Results

### Patients

A total of 12 637 patients were included in the pooled analysis (3297 from SUSTAIN 6 and 9340 from LEADER), with 6316 patients in the combined semaglutide/liraglutide group (826, 822, and 4668 patients in the semaglutide 0.5 mg, semaglutide 1.0 mg, and liraglutide 1.8 mg group, respectively) and 6321 patients in the combined placebo group. The median durations of follow-up (after randomization) were 2.1 and 3.8 years for SUSTAIN 6 and LEADER, respectively.^6,7^ Patient characteristics according to albuminuria and eGFR and treatment allocation are presented in the Table. At baseline, a total of 3063 (24.2%) patients had an eGFR <60 mL/min/1.73 m^2^ and 4726 (38.2%) patients had elevated albuminuria, either microalbuminuria (27.0%) or macroalbuminuria (11.2%).

**Table.**
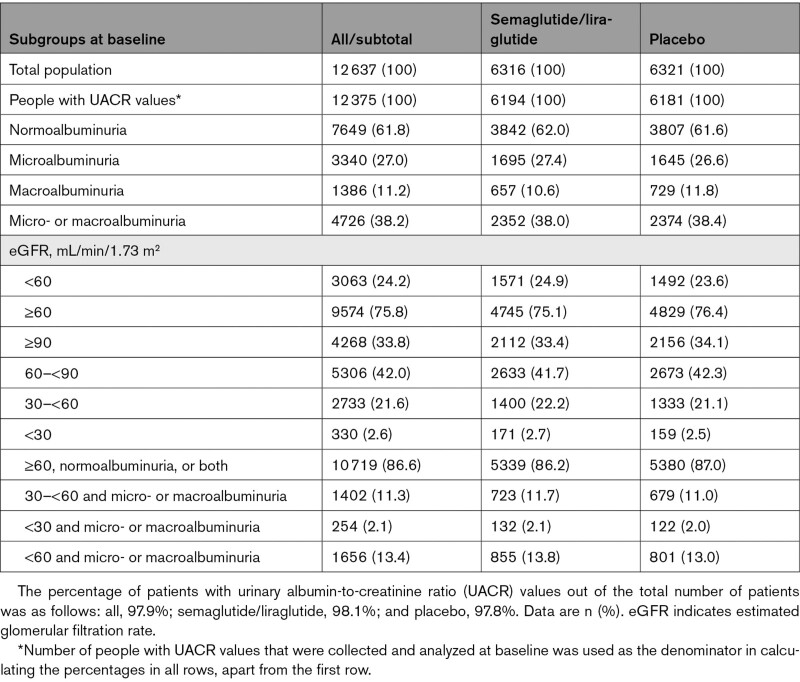
Baseline Albuminuria and eGFR Characteristics, by Treatment Group

### Effects on Albuminuria

**Figure 1.**
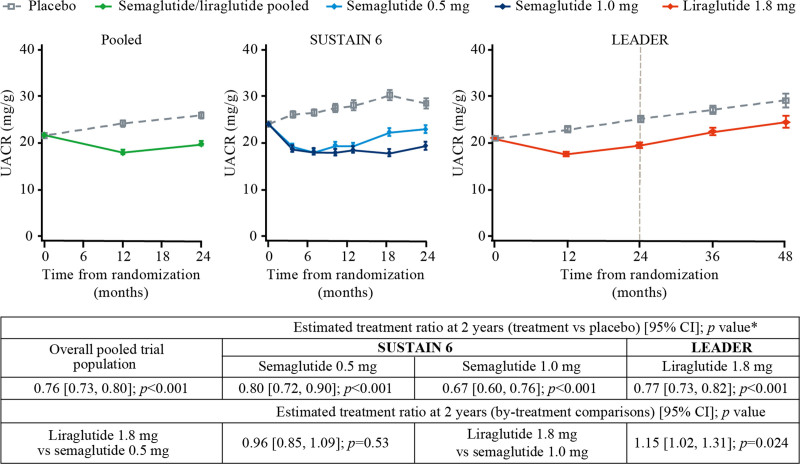
**Effects of once-weekly semaglutide and once-daily liraglutide versus placebo on albuminuria over time.** *Estimated geometric mean ratio calculated for each active treatment group versus the respective placebo group. Geometric mean values of albuminuria over time with semaglutide and liraglutide by trial as compared with placebo were estimated using a mixed model for repeated measures with an unstructured covariance matrix for repeated measures. Urinary albumin-to-creatinine ratio (UACR) was included as a dependent variable (which was log-transformed) with treatment and visits as fixed factors and baseline UACR as a covariate (log-transformed). Pooled analyses were done using the same model with trial also included as a fixed factor. LEADER indicates Liraglutide Effect and Action in Diabetes: Evaluation of Cardiovascular Outcome Results; and SUSTAIN 6, Trial to Evaluate Cardiovascular and Other Long-Term Outcomes With Semaglutide in Subjects With Type 2 Diabetes.

In the pooled analysis, semaglutide/liraglutide treatment lowered albuminuria from baseline to 2 years after randomization by 24% (placebo-corrected geometric mean ratio of relative change from baseline) compared with placebo (95% CI, 20%–27%; *P*<0.001; Figure 1). This was driven by data from liraglutide, given the larger number of patients from LEADER than from SUSTAIN 6. The magnitude of reduction, however, was modified by baseline level of albuminuria: normoalbuminuria (20% [95% CI, 15%–25%]), microalbuminuria (31% [95% CI, 25%–37%]), and macroalbuminuria (19% [95% CI, 7%–30%]; *P*_interaction_=0.021; Figure S1).

Both semaglutide and liraglutide lowered albuminuria compared with placebo (Figure 1). At 2 years after randomization, on the basis of the placebo-corrected geometric mean ratios of relative change from baseline, semaglutide 0.5 mg lowered albuminuria by 20% compared with placebo (95% CI, 10%–28%; *P*<0.001) and the 1.0 mg dose lowered albuminuria by 33% compared with placebo (95% CI, 24%–40%; *P*<0.001). At 2 years after randomization, albuminuria was 23% lower in liraglutide-treated patients compared with placebo (95% CI, 18%–27%; *P*<0.001). The effect of the semaglutide 1.0 mg dose was statistically greater than that of liraglutide (*P*=0.024) at 2 years after randomization.

### Effect on eGFR Slope

**Figure 2.**
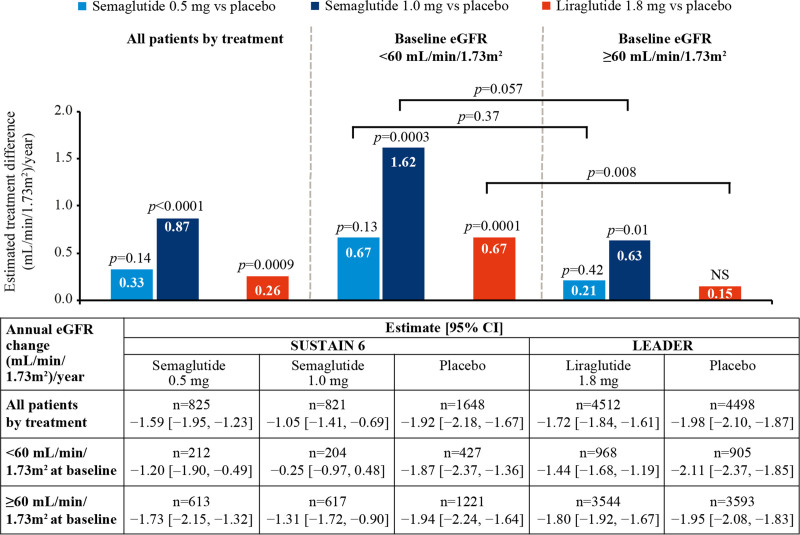
**Effects of once-weekly semaglutide and once-daily liraglutide versus placebo on average annual eGFR decline.** Effects of once-weekly semaglutide and once-daily liraglutide versus placebo on average annual estimated glomerular filtration rate (eGFR) decline (slope) in all patients and according to baseline eGFR. N is the number of patients whose samples/measures were available at the point of analysis. Slope analyses were performed on the intent-to-treat population. Slope analyses of eGFR were performed using a random-slope model by trial with change from baseline as dependent variable and baseline value and time (in years) as covariate and treatment as a fixed factor and the interaction between treatment and time. Patient-specific intercepts and time as random effects assuming a bivariate normal distribution for these effects were included in the model. Analyses by subgroups were performed by including the respective subgroups as a fixed factor and the interaction with treatment. Data shown were averaged over 2 years. LEADER indicates Liraglutide Effect and Action in Diabetes: Evaluation of Cardiovascular Outcome Results; and SUSTAIN 6, Trial to Evaluate Cardiovascular and Other Long-Term Outcomes With Semaglutide in Subjects With Type 2 Diabetes.

The average slope in eGFR change from baseline was comparable in the placebo arms of SUSTAIN 6 (average, −1.92 [95% CI, −2.18 to −1.67 mL/min/1.73 m^2^/y]) and LEADER (average, −1.98 [95% CI, −2.10 to −1.87 mL/min/1.73 m^2^/y]) in the overall population, and this was broadly consistent at different levels of baseline kidney function (Figure 2).

In the overall population, patients randomized to semaglutide 0.5 mg had a nonsignificant reduction in eGFR slope versus placebo (difference 0.33 mL/min/1.73 m^2^/y; *P*=0.14), whereas randomization to semaglutide 1.0 mg slowed kidney function loss by 0.87 mL/min/1.73 m^2^/y versus placebo (*P*<0.0001; Figure 2). Kidney function in patients randomized to liraglutide declined an average of 0.26 mL/min/1.73 m^2^/y slower compared with placebo (*P*<0.001).

Baseline eGFR was found to impact the effects of semaglutide and liraglutide on eGFR slope, with the largest effect observed in patients receiving semaglutide 1.0 mg with an eGFR <60 mL/min/1.73 m^2^/y at baseline. Patients in this subgroup lost 1.62 mL/min/1.73 m^2^/y less kidney function versus patients receiving placebo, whereas patients with baseline eGFR ≥60 mL/min/1.73 m^2^/y lost 0.63 mL/min/1.73 m^2^/y less versus placebo (*P*<0.001; *P*_interaction_=0.06). The difference was 0.67 versus 0.21 mL/min/1.73 m^2^/y (*P*_interaction_=0.37) for patients randomized to semaglutide 0.5 mg with baseline eGFR <60 or >60 mL/min/1.73 m^2^/y, respectively. Patients receiving liraglutide with baseline eGFR <60 mL/min/1.73 m^2^ lost 0.67 mL/min/1.73 m^2^/y less kidney function than patients receiving placebo, whereas patients with baseline eGFR ≥60 mL/min/1.73 m^2^ lost 0.15 mL/min/1.73 m^2^/y (*P*_interaction_=0.008; Figure 2).

The results were similar when the total loss of kidney function over 2 years after randomization of all treatments was considered (Figure S2).

The effect of semaglutide on eGFR slope compared with placebo was not clearly modified by level of albuminuria at baseline. Semaglutide 0.5 mg effect on eGFR slope compared with placebo ranged from 0.59 mL/min/1.73 m^2^ (95% CI, −0.76 to 1.94 mL/min/1.73 m^2^) in the subgroup with normoalbuminuria to 0.29 mL/min/1.73 m^2^ (95% CI, −2.16 to 2.74 mL/min/1.73 m^2^) in the subgroup with macroalbuminuria (*P*_interaction_=0.98) at 2 years after randomization. Semaglutide 1.0 mg effect on eGFR slope compared with placebo ranged from 1.48 mL/min/1.73 m^2^ (95% CI, 0.15–2.80 mL/min/1.73 m^2^) in the subgroup with normoalbuminuria to 2.33 mL/min/1.73 m^2^ (95% CI, −0.19 to 4.85 mL/min/1.73 m^2^) in the subgroup with macroalbuminuria (*P*_interaction_=0.84). The effect of liraglutide on eGFR slope compared with placebo appeared to be modified by the degree of albuminuria at baseline, with eGFR slope differences ranging from 0.20 mL/min/1.73 m^2^ (95% CI, −0.17 to 0.58 mL/min/1.73 m^2^) with normoalbuminuria to 1.64 mL/min/1.73 m^2^ (95% CI, 0.68–2.60 mL/min/1.73 m^2^) with macroalbuminuria at 2 years after randomization (*P*_interaction_=0.023; Figure S2).

### Effect on Persistent Reduction in eGFR

**Figure 3.**
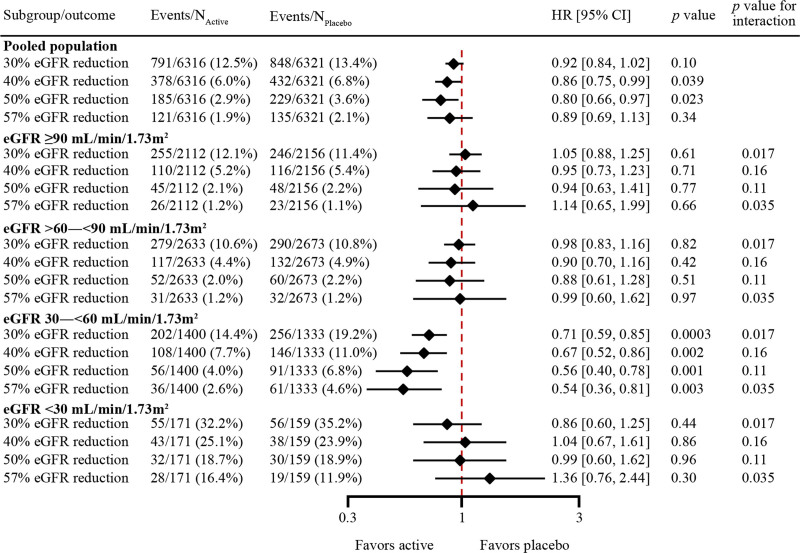
**Effects of semaglutide and liraglutide versus placebo on time to the first persistent reduction in eGFR in the pooled population and subgroups according to eGFR at baseline.** Effects of semaglutide and liraglutide versus placebo on time to the first persistent reduction in estimated glomerular filtration rate (eGFR) of 30%, 40%, 50%, and 57% from baseline in the pooled population and subgroups according to eGFR (mL/min/1.73 m^2^) at baseline. Time to persistent reduction of eGFR from baseline was analyzed independently from each other. Subgroup analyses were performed by including subgroup as a fixed factor and the interaction between subgroup and treatment. HR indicates hazard ratio.

The overall effect of semaglutide/liraglutide (pooled) versus placebo on the risk of persistent reductions in eGFR using a range of clinically relevant thresholds is shown in Figure 3. In the overall population, persistent 40% and 50% reductions in eGFR occurred less frequently in patients receiving semaglutide/liraglutide compared with placebo (HR, 0.86 [95% CI, 0.75–0.99]; *P*=0.039 and HR, 0.80 [95% CI, 0.66–0.97]; *P*=0.023, respectively; Figure 3). The risk of reaching a persistent 30% eGFR reduction (HR, 0.92 [95% CI, 0.84–1.02]; *P*=0.10) or a persistent 57% reduction (HR, 0.89 [95% CI, 0.69–1.13]; *P*=0.34) was not significantly reduced but showed similar directional results (Figure 3).

In subgroups of patients with an eGFR of 30 to <60 mL/min/1.73 m^2^ at baseline, persistent reduction in eGFR for all thresholds occurred in fewer patients in the semaglutide/liraglutide group compared with the placebo group, with a trend to greater effect sizes as eGFR thresholds increased. The HR (95% CI; percentage semaglutide/liraglutide vs placebo) values were as follows: 30% reduction: 0.71 (0.59–0.85), *P*=0.0003 (14.4% vs 19.2%); 40% reduction: 0.67 (0.52–0.86), *P*=0.0017 (7.7% vs 11.0%); 50% reduction: 0.56 (0.40–0.78), *P*=0.0006 (4.0% vs 6.8%); 57% reduction: 0.54 (0.36–0.81), *P*=0.003 (2.6% vs 4.6%; Figure 3). The effect sizes appeared larger in subgroups of patients with eGFR 30 to <60 mL/min/1.73 m^2^ at baseline compared with other subgroups (eGFR ≥90, 60−<90, and <30 mL/min/1.73 m^2^ at baseline), especially for 30% and 57% reduction thresholds (*P*_interaction_=0.017 and 0.035, respectively; Figure 3). On a continuous eGFR scale, the treatment effect associated with semaglutide/liraglutide versus placebo increased as baseline eGFR decreased (Figure S3).

**Figure 4.**
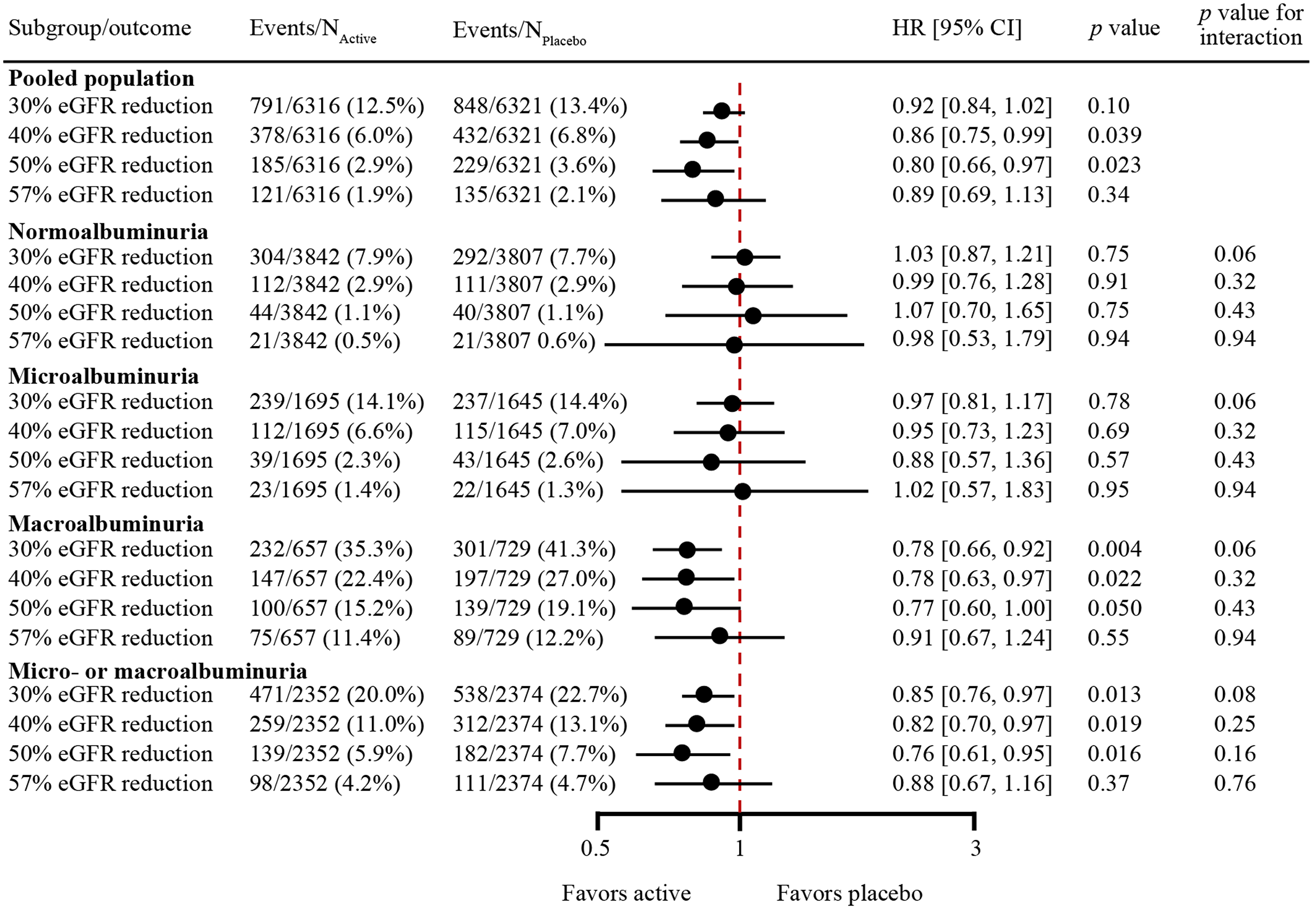
**Effects of semaglutide and liraglutide versus placebo on time to the first persistent reduction in eGFR in the pooled population and subgroups defined by the level of albuminuria at baseline.** Effects of semaglutide/liraglutide versus placebo on time to the first persistent reduction in estimated glomerular filtration rate (eGFR) of 30%, 40%, 50%, and 57% from baseline in the pooled population and subgroups defined by the level of albuminuria at baseline. Time to persistent reduction of eGFR from baseline was analyzed independently from each other. Subgroup analyses were performed by including subgroup as a fixed factor and the interaction between subgroup and treatment. HR indicates hazard ratio.

The results were broadly consistent for subgroups on the basis of baseline albuminuria (Figure 4), with separately statistically significant reductions in the risk of persistent 30%, 40%, and 50% reductions in eGFR with semaglutide/liraglutide compared with placebo in subgroups of patients with macroalbuminuria (HRs ranged from 0.78 [95% CI, 0.66–0.92]; *P*=0.004 for 30% reduction in eGFR to 0.77 [95% CI, 0.60–1.00]; *P*=0.050 for 50% reduction in eGFR) and microalbuminuria or macroalbuminuria at baseline (HRs ranged from 0.85 [95% CI, 0.76–0.97]; *P*=0.013 for 30% reduction in eGFR to 0.76 [95% CI, 0.61–0.95]; *P*=0.016 for 50% reduction in eGFR).

Similar patterns were observed in subgroups of patients stratified on the basis of a combination of baseline eGFR and albuminuria criteria (Figure S4). The effect of semaglutide/liraglutide therapy on persistent reduction in eGFR for all thresholds appeared to be larger in subgroups of patients with eGFR 30 to <60 mL/min/1.73 m^2^ and microalbuminuria or macroalbuminuria at baseline than in subgroups with baseline eGFR of ≥60 mL/min/1.73 m^2^ or normoalbuminuria as well as subgroups with eGFR <30 mL/min/1.73 m^2^ and microalbuminuria or macroalbuminuria at baseline.

When persistent reductions in eGFR were combined with kidney failure and kidney death to form 3 composite outcomes, similar findings were observed (Figures S5−S7).

## Discussion

This post hoc analysis comprising 12 637 patients with T2D suggests that semaglutide and liraglutide have kidney-protective effects. Both semaglutide and liraglutide lowered albuminuria, slowed eGFR decline, and reduced the risk of substantial loss of kidney function at different thresholds. The effects of semaglutide and liraglutide on kidney function appear to be greater in subgroups of patients with preexisting kidney disease, defined by reduced eGFR, increased albuminuria, or the combination of both. The data also suggest differences between agents, with the largest magnitude of protective effects observed for semaglutide 1.0 mg.

The likelihood of a kidney-protective effect with GLP-1 RA therapy is supported by the results of other studies. In AWARD-7 (A Study Comparing Dulaglutide With Insulin Glargine on Glycemic Control in Participants With Type 2 Diabetes and Moderate or Severe Chronic Kidney Disease; URL: https://www.clinicaltrials.gov; Unique identifier: NCT01621178), dulaglutide was found to slow eGFR decline compared with insulin glargine in people with advanced chronic kidney disease,^22^ with identical control of HbA1c in both groups. Dulaglutide also reduced the risk of substantial loss of kidney function compared with placebo in the REWIND trial (Researching Cardiovascular Events With a Weekly Incretin in Diabetes; URL: https://www.clinicaltrials.gov; Unique identifier: NCT01394952), which examined kidney outcomes as key secondary end points.^9^ ELIXA (Evaluation of Cardiovascular Outcomes in Patients With Type 2 Diabetes After Acute Coronary Syndrome During Treatment With AVE0010 [Lixisenatide]; URL: https://www.clinicaltrials.gov; Unique identifier: NCT01147250)^23^ and EXSCEL (Exenatide Study of Cardiovascular Event Lowering Trial; URL: https://www.clinicaltrials.gov; Unique identifier: NCT01144338)^24^ monitored kidney outcomes only as adverse events and failed to demonstrate benefits for kidney function per se, despite moderate lowering albuminuria or lower incidence of new microalbuminuria with lixisenatide and exenatide, respectively. In a post hoc analysis of EXSCEL, the positive effect of exenatide on the eGFR slope was more pronounced in patients with higher albuminuria at baseline versus patients with normal albuminuria.^25^ Exenatide, in a post hoc analysis, also reduced composites of either 40% or 30% eGFR decline and kidney replacement therapy.^24^ Variability in effects of GLP-1 RAs on cardiovascular outcomes has also been demonstrated,^13^ highlighting the inability to generalize effects across different members of the drug class at doses that generally lead to comparable lowering of HbA1c. These variable cardiovascular and kidney benefits may be attributable to differences in trial conduct or to the medicines or their degradation products potentially having favorable effects with human-based, but not exendin-based, GLP-1 RAs in T2D.^13,26^

The mechanisms for the potential protective effect of semaglutide and liraglutide on the kidneys are uncertain. Possible contributing mechanisms include natriuresis, oxidative stress reduction, reduced inflammation and fibrosis, and hemodynamic effects.^13,27,28^ Alternative possible mechanisms include the indirect modification of kidney risk through lowering glucose levels, body weight, and blood pressure.^17,29,30^ However, the latter indirect effects likely only play a minor role according to mediation analyses, and the effects on blood pressure are modest.^22,31,32^ In a recent post hoc analysis of LEADER and SUSTAIN 6, HbA1c mediated 25% and 26%, respectively, of the kidney protective effect associated with liraglutide and semaglutide, whereas the mediatory effects of systolic blood pressure and body weight were lower at 9% and 22% and 9% and 0%, respectively.^32^ A previous post hoc analysis of LEADER demonstrated that baseline HbA1c did not influence the kidney-protective effect of liraglutide.^15^ As previously mentioned, dulaglutide and insulin lowered HbA1c to the same extent in AWARD-7. Despite this, patients receiving dulaglutide had a significantly lower eGFR decrease compared with insulin, again suggesting that HbA1c alone does not drive the kidney-protective effects observed with GLP-1 RAs.^22^ The reason why the benefits seen might be greater in people with established kidney disease is uncertain.

The larger magnitude of effect of the semaglutide 1.0 mg dose compared with both the semaglutide 0.5 mg dose and liraglutide on both albuminuria and eGFR, along with likely benefits on substantial reductions in kidney function, suggests that this agent in particular might have an important role in protecting kidney function in diabetes. This may be especially relevant in people with existing DKD. Although these post hoc analyses of secondary outcomes are promising, a specific, a sufficiently powered trial aiming to assess the effects of semaglutide/liraglutide on kidney outcomes in people with diabetes and kidney disease is needed. FLOW (A Research Study to See How Semaglutide Works Compared to Placebo in People With Type 2 Diabetes and Chronic Kidney Disease; URL: https://www.clinicaltrials.gov; Unique identifier: NCT03819153) specifically addresses the question regarding slowing progression of DKD. The trial will enroll 3508 patients with T2D and kidney disease to either semaglutide 1.0 mg subcutaneously or matching placebo. The primary outcome is a composite of kidney failure (dialysis or transplantation and persistent eGFR <15 mL/min/1.73 m^2^), a persistent ≥50% reduction in eGFR, and kidney or cardiovascular death. FLOW will provide a definitive assessment of the kidney protection suggested in this analysis. In addition, kidney outcomes in patients treated with semaglutide will be investigated as secondary end points in both SOUL (A Heart Disease Study of Semaglutide in Patients With Type 2 Diabetes; URL: https://www.clinicaltrials.gov; Unique identifier: NCT03914326) and SELECT (Semaglutide Effects on Heart Disease and Stroke in Patients With Overweight or Obesity; URL: https://www.clinicaltrials.gov; Unique identifier: NCT03574597).

The main strength of our analysis is the incorporation of 2 large randomized clinical trials that prospectively defined adjudicated kidney outcomes as key secondary outcomes. There are also some limitations. This is an exploratory analysis and was not prespecified. SUSTAIN 6 and LEADER were not originally powered to evaluate kidney outcomes and included patients with relatively low kidney risk at baseline. We did not take into account competing risk from terminal events (eg, cardiovascular death), which could favor especially the liraglutide arm versus placebo, as for this end point there was a 22% risk reduction compared with placebo overall.^6,7^ Whereas persistent reduction in eGFR was defined as the first occurrence of reduction confirmed by a subsequent measurement, patients with eGFR reduction whose subsequent eGFR could not be measured (either because of a fatal event or an eGFR reduction at the last schedule visit) were included in the analysis. This likely influenced the sustained eGFR reduction analysis (Table S1).

This pooled analysis suggests a kidney-protective effect of semaglutide and liraglutide. This effect seems to be more pronounced in people with DKD. FLOW will prospectively test the effect of semaglutide in this patient group and may identify an additional therapeutic strategy for people with T2D and kidney disease.

## Article Information

### Acknowledgments

The authors thank the trial personnel and participants.

### Sources of Funding

The study was funded by Novo Nordisk A/S. The authors thank Jin Heppell, PhD, Aneela Majid, PhD, and Izabel James, MBBS, of Ashfield MedComms, an Ashfield Health company (funded by Novo Nordisk A/S), for incorporation of comments and editorial assistance.

### Disclosures

Dr Shaman reports no conflicts of interest. Dr Bain reports personal fees from AstraZeneca (modest), Boehringer Ingelheim (modest), Eli Lilly (modest), Merck Sharp & Dohme (modest), Novo Nordisk (significant), and Sanofi-Aventis (modest). Dr Bakris reports monies paid to his institution (University of Chicago Medicine) for steering committee participation and consulting fees from Merck, Relypsa, Vifor, and AstraZeneca (all modest). Dr Buse reports contracted consulting fees and travel support for contracted activities paid to the University of North Carolina by Adocia, AstraZeneca, Dance Biopharm, Dexcom, Eli Lilly, Fractyl, GI Dynamics, Intarcia Therapeutics, Lexicon, MannKind, Metavention, NovaTarg, Novo Nordisk, Orexigen, PhaseBio, Sanofi, Senseonics, vTv Therapeutics, and Zafgen; receives grant support from AstraZeneca, Eli Lilly, Intarcia Therapeutics, Johnson & Johnson, Lexicon, Medtronic, NovaTarg, Novo Nordisk, Sanofi, Theracos, Tolerion, and vTv Therapeutics; is a consultant to Cirius Therapeutics Inc, CSL Behring, Mellitus Health, Neurimmune AG, Pendulum Therapeutics, and Stability Health; holds stock/options in Mellitus Health, Pendulum Therapeutics, PhaseBio, and Stability Health; and receives grant support from the National Institutes of Health (UL1TR002489 and P30DK124723). Dr Idorn is a Novo Nordisk employee and stockholder (modest). Dr Mahaffey reports research grants from the American Heart Association, Apple, Inc, Bayer, California Institute Regenerative Medicine, Eidos, Ferring, Gilead, Google (Verily), Idorsia‚ Johnson & Johnson, Luitpold, PAC-12‚ Precordior‚ and Sanifit; consulting or other service fees (including CME) from Amgen, Applied Therapeutics, AstraZeneca, Bayer, CSL Behring, Elsevier, Fibrogen‚ Inova, Johnson & Johnson, Lexicon‚ Myokardia, Novartis, Novo Nordisk, Otsuka, PhaseBio, Portola, Sanofi, and Theravance (significant for the Novo Nordisk relationship). Dr Mann has received speaker honoraria from AstraZeneca, Medice, Novartis, Novo Nordisk, Roche, and Vifor Pharma; research support from the European Union, Population Health Research Institute, AstraZeneca, Bayer, Boehringer Ingelheim, Idorisia, and Novo Nordisk; and consultation fees from AstraZeneca, Bayer, Merck, Novo Nordisk, and Vifor Pharma (all modest, except support from the European Union [significant]). Dr Nauck has served on advisory boards or consulted for AstraZeneca (modest), Boehringer Ingelheim (modest), Eli Lilly & Co (significant), Menarini/Berlin Chemie (modest), Merck, Sharp & Dohme (significant), and Novo Nordisk (significant); his institution has received grant support from AstraZeneca, Boehringer Ingelheim, Eli Lilly & Co, GlaxoSmithKline, Intarcia, Menarini/Berlin-Chemie, Merck, Sharp & Dohme, Novartis Pharma, and Novo Nordisk A/S; he has served on speakers’ bureaus of AstraZeneca (modest), Boehringer Ingelheim (modest), Eli Lilly & Co (modest), GlaxoSmithKline (modest), Menarini/Berlin Chemie (modest), Sun Pharma (modest), Merck, Sharp & Dohme (significant), and Novo Nordisk A/S (significant). Dr Rasmussen is a Novo Nordisk employee and stockholder (significant). Dr Rossing is on the steering committee of the following clinical trials: CADA-DIA (Bayer), Fidelio (Bayer), Figaro (Bayer), DAPA-CKD (Astra Zeneca), and FLOW (Novo-Nordisk); and The Steno Diabetes Center Copenhagen has received fees for consultancy and/or speaking from Astellas, AstraZeneca, Bayer, Boehringer Ingelheim, Gilead, Eli Lilly, Mundi, Novo Nordisk, and Sanofi Aventis (all modest). Dr Wolthers is a Novo Nordisk employee and stockholder. Dr Zinman has received consulting fees from Merck, Novo Nordisk, Sanofi-Aventis, Eli Lilly, AstraZeneca, Janssen, and Boehringer Ingelheim (all modest). Dr Perkovic reports consultancy for AbbVie, Inc, Amgen, AstraZeneca, Bayer Healthcare, Boehringer Ingelheim, Chinook Therapeutics, Dimerix Bioscience, Gilead Sciences Inc, GlaxoSmithKline, Janssen Global Services, LLC, Metavant Sciences, Mundipharma, Novartis Pharma, Novo Nordisk AS, Travere Therapeutics, Inc, and Tricida; and other for George Clinical, Mitsubishi Tanabe Pharma Corporation, and UpToDate. Drs Idorn, Rasmussen, and Wolthers verified the underlying data and Dr Rasmussen performed the statistical analyses. Drs Shaman and Perkovic wrote the first draft of the manuscript. Dr Perkovic had final responsibility for the decision to submit for publication. All authors contributed significantly to the design and conduct of the study and acquisition of clinical data, reviewed and interpreted the data, were involved in drafting and critically revising the manuscript, approved the final version of the manuscript, and take full responsibility for the content.

### Supplemental Material

Table S1

Figures S1–S7

## Supplementary Material


