# Supplementary material for: Effect of the Glucagon-Like Peptide-1 Receptor Agonists Semaglutide and Liraglutide on Kidney Outcomes in Patients With Type 2 Diabetes: Pooled Analysis of SUSTAIN 6 and LEADER
Source: Circulation. 2021 Dec 14;145(8):575–85. doi: 10.1161/CIRCULATIONAHA.121.055459 (PMC8860212; doi:10.1161/CIRCULATIONAHA.121.055459)
Supplement: Supplementary file 1 [file cir-145-575-s001.pdf]

SUPPLEMENTAL MATERIAL

Effect of the glucagon-like peptide-1 receptor agonists semaglutide and liraglutide on kidney outcomes in patients with type 2 diabetes: a pooled analysis of SUSTAIN 6 and LEADER trials

Supplementary Table 1. Sensitivity analysis testing the effects of semaglutide/liraglutide versus placebo on time to the first persistent reduction in eGFR of 30%, 40%, 50% and 57% from baseline in patients with a confirmatory subsequent eGFR measurement.

**Sensitivity analysis**	**Pooled population**			**eGFR subgroup (30–<60 mL/min/1.73 m^2)**			
Outcome	Events/N_{Active} (%)	HR [95% CI]	*P* value	Events/N_{Active}	HR [95% CI]	*P* value	*P* value for interaction
Sustained 30% eGFR reduction	461/6316 (7.3%)	0.94 [0.83, 1.07]	0.33	112/1400 (8.0%)	0.68 [0.53, 0.87]	0.0022	0.02
Sustained 40% eGFR reduction	198/6316 (3.1%)	0.89 [0.74, 1.08]	0.25	58/1400 (4.1%)	0.75 [0.53, 1.06]	0.10	0.78
Sustained 50% eGFR reduction	95/6316 (1.5%)	0.86 [0.65, 1.13]	0.27	31/1400 (2.2%)	0.65 [0.41, 1.02]	0.06	0.53
Sustained 57% eGFR reduction	66/6316 (1.0%)	0.96 [0.69, 1.35]	0.83	24/1400 (1.7%)	0.82 [0.47, 1.42]	0.47	0.88

Participants required two consecutive values meeting the endpoint to be considered responders. If the condition was only fulfilled at the last scheduled visit, it was not counted as a sustained reduction.
Analysis was based on a Cox proportional hazards model with treatment as a fixed factor and stratified by study.
HR compared liraglutide/semaglutide with placebo. HR, hazard ratio.

Supplementary Figure 1. Effects of semaglutide/liraglutide versus placebo on urinary albumin-to-creatinine ratio in the pooled population and subgroups defined by the level of albuminuria at baseline.

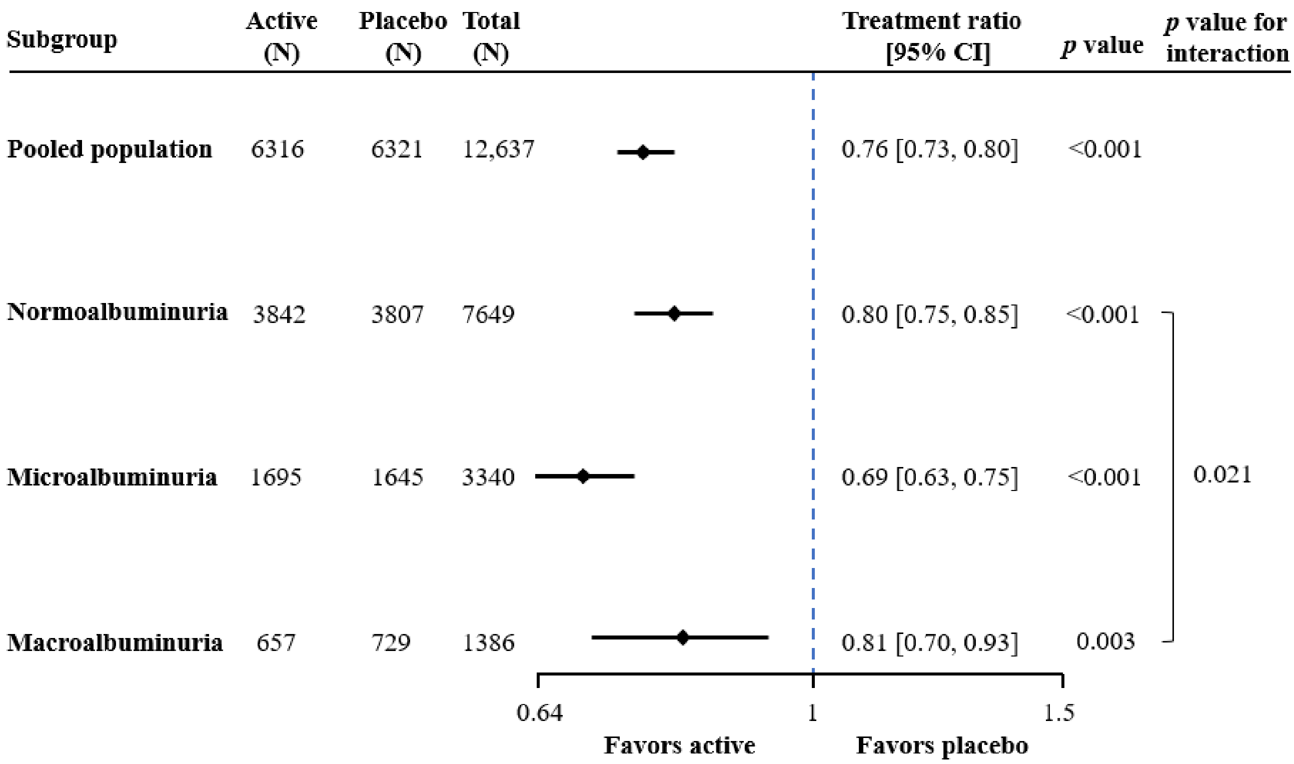

For the pooled population, a MMRM with treatment, study, visits and baseline albuminuria (log-transformed) all nested within patient included as factors. An unstructured covariance matrix for repeated measures was used. For the stratified albuminuria categories, the same model was used with baseline albuminuria category and treatment by baseline albuminuria category interaction included as additional factors.
CI, confidence interval; MMRM, mixed model for repeated measures.

Supplementary Figure 2. Effect of semaglutide and liraglutide versus placebo on the annual eGFR slope at 2 years visit in the overall population and subgroups with pre-existing DKD defined by the level of albuminuria and eGFR at baseline.

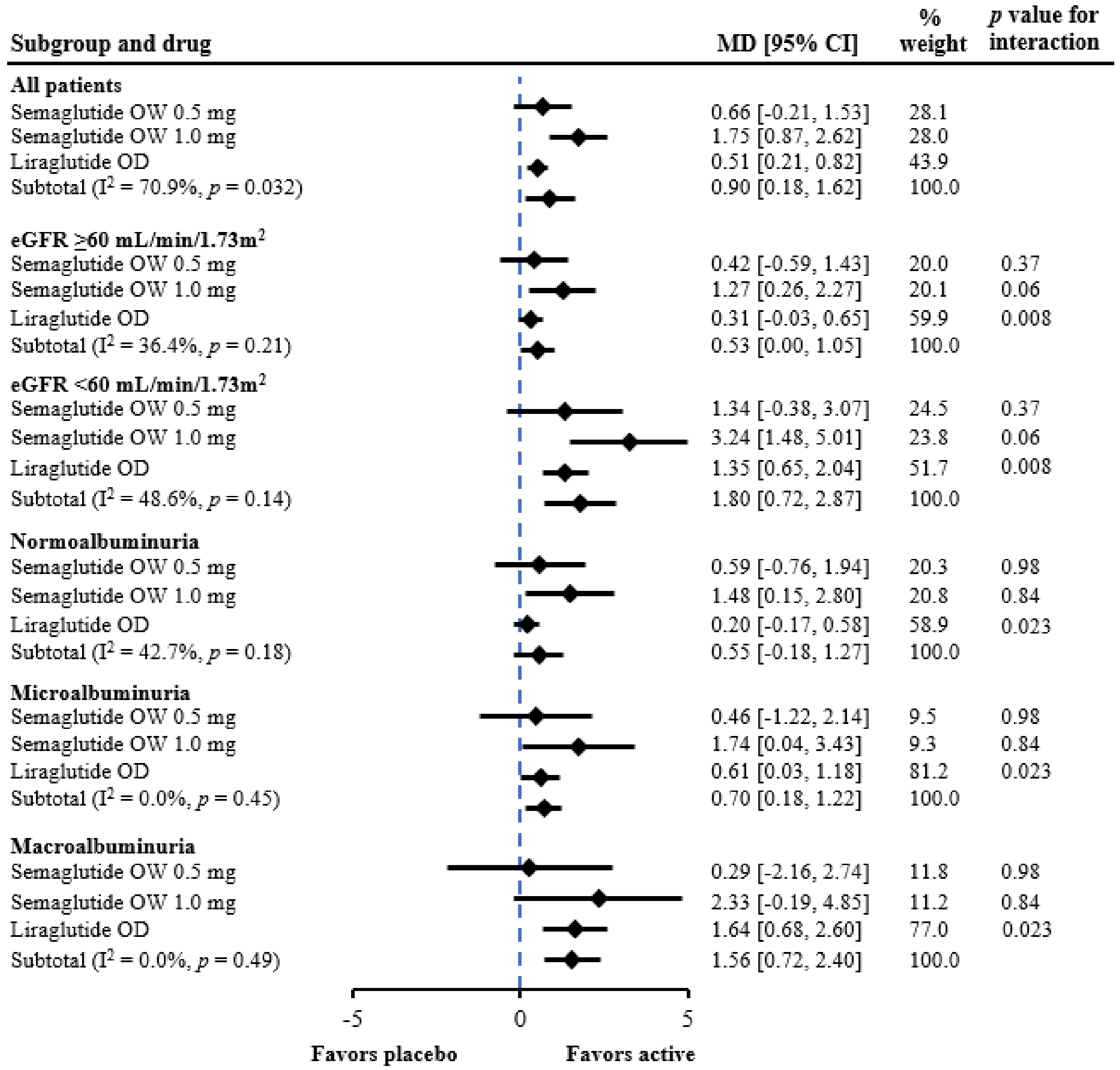

Slope analyses of eGFR were performed using a random-slope model by trial with change from baseline as dependent variable and baseline value and time (in years) as covariate, and treatment as a fixed factor and the interaction between treatment and time. Patient-specific intercepts and time as random effects assuming a bivariate normal distribution for these effects were included in the model. Analyses by subgroups were performed by including the respective subgroups as a fixed factor and the interaction with treatment. CI, confidence interval; DKD, diabetic kidney disease; eGFR, estimated glomerular filtration rate; MD, mean difference (mL/min/1.73m^2) OW semaglutide or OD liraglutide versus placebo; OD, once-daily; OW, once-weekly.

Supplementary Figure 3. Effects of semaglutide/liraglutide versus placebo (as a hazard ratio) on the time to first sustained 30%, 40%, 50% and 57% eGFR reduction according to eGFR (mL/min/1.73m^2) at baseline

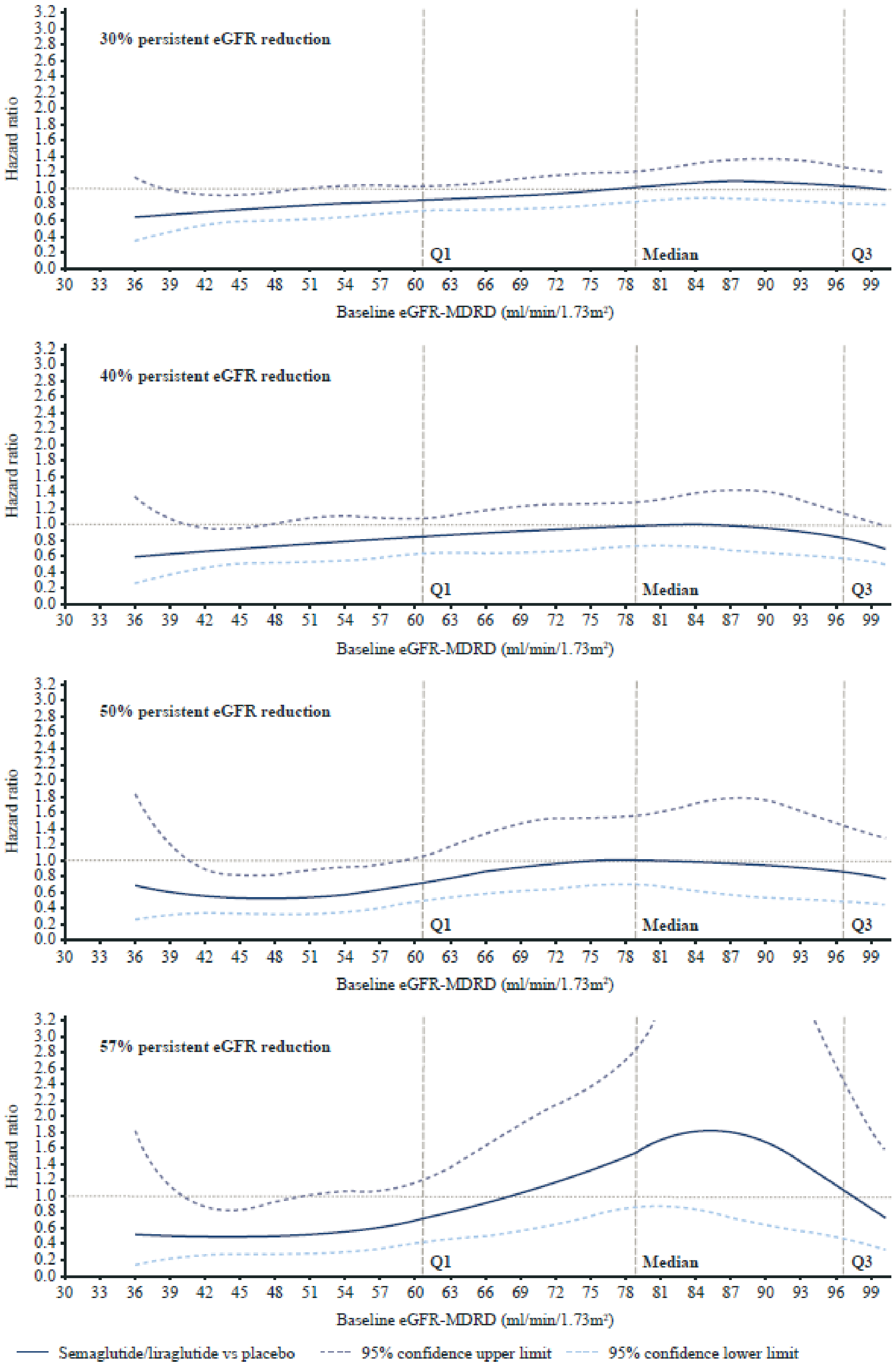

Effects of semaglutide/liraglutide versus placebo on the time to first sustained reduction in eGFR according to eGFR at baseline was calculated using a quadratic spline Cox regression model.

Supplementary Figure 4. Effects of semaglutide/liraglutide versus placebo on time to first persistent reduction in eGFR of 30%, 40%, 50% and 57% from baseline, in the pooled population and subgroups with pre-existing DKD defined by eGFR (mL/min/1.73m^2) and level of albuminuria at baseline.

Subgroup/outcome	Events/N_{Active}	Events/$N_{Placebo}$	HR [95% CI]	*p* value	*p* value for interaction
**Pooled population**					
30% eGFR reduction	791/6316 (12.5%)	848/6321 (13.4%)	0.92 [0.84, 1.02]	0.10	
40% eGFR reduction	378/6316 (6.0%)	432/6321 (6.8%)	0.86 [0.75, 0.99]	0.039	
50% eGFR reduction	185/6316 (2.9%)	229/6321 (3.6%)	0.80 [0.66, 0.97]	0.023	
57% eGFR reduction	121/6316 (1.9%)	135/6321 (2.1%)	0.89 [0.69, 1.13]	0.34	
**eGFR ≥60 mL/min/1.73m^2 or normoalbuminuria**					
30% eGFR reduction	580/5423 (10.7%)	595/5488 (10.8%)	0.98 [0.88, 1.10]	0.76	0.004
40% eGFR reduction	245/5423 (4.5%)	272/5488 (5.0%)	0.90 [0.76, 1.07]	0.25	0.049
50% eGFR reduction	101/5423 (1.9%)	119/5488 (2.2%)	0.85 [0.65, 1.11]	0.24	0.10
57% eGFR reduction	58/5423 (1.1%)	62/5488 (1.1%)	0.94 [0.66, 1.34]	0.73	0.034
**eGFR 30—<60 mL/min/1.73m^2 & micro- or macroalbuminuria**					
30% eGFR reduction	152/723 (21.0%)	197/679 (29.0%)	0.65 [0.53, 0.81]	<0.0001	0.004
40% eGFR reduction	89/723 (12.3%)	121/679 (17.8%)	0.63 [0.48, 0.83]	0.001	0.049
50% eGFR reduction	51/723 (7.1%)	78/679 (11.5%)	0.57 [0.40, 0.81]	0.002	0.097
57% eGFR reduction	34/723 (4.7%)	53/679 (7.8%)	0.56 [0.37, 0.87]	0.009	0.034
**eGFR <30 mL/min/1.73m^2 & micro- or macroalbuminuria**					
30% eGFR reduction	52/132 (39.4%)	52/122 (42.6%)	0.89 [0.61, 1.31]	0.56	0.004
40% eGFR reduction	42/132 (31.8%)	37/122 (30.3%)	1.07 [0.69, 1.67]	0.75	0.049
50% eGFR reduction	32/132 (24.2%)	30/122 (24.6%)	1.02 [0.62, 1.68]	0.93	0.10
57% eGFR reduction	28/132 (21.2%)	19/122 (15.6%)	1.42 [0.79, 2.55]	0.24	0.034

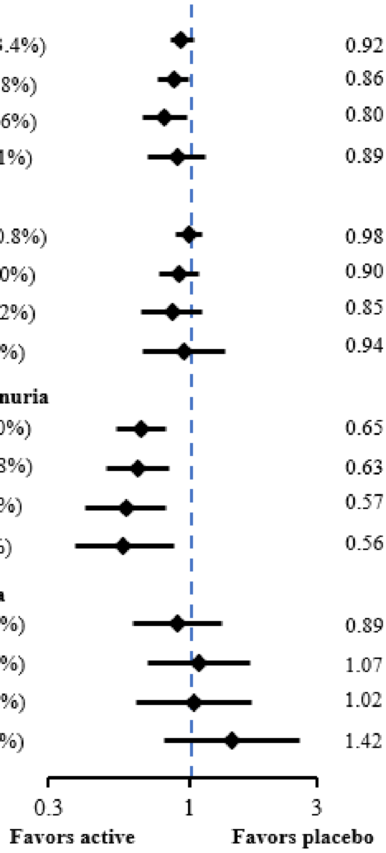

Time to first event analyses were performed using Cox proportional hazard models with pooled treatment as a fixed factor and stratified by trial. Patients without respective events were censored at death or the end of follow-up, whichever came first. Time to persistent reduction of eGFR from baseline (30%, 40%, 50% and 57%) was analysed independently from each other. Subgroup analyses were performed by including subgroup as a fixed factor and the interaction between subgroup and treatment. CI, confidence interval; DKD, diabetic kidney disease; eGFR, estimated glomerular filtration rate; HR, hazard ratio.

Supplementary Figure 5. Effects of semaglutide/liraglutide versus placebo on the risk of composite kidney outcomes (kidney death, kidney failure or proportional eGFR decline [40%, 50% and 57%]), in the pooled population and subgroups defined by eGFR (mL/min/1.73m^2) at baseline.

Subgroup/outcome	Events/N_{Active}	Events/$N_{Placebo}$	HR [95% CI]	*p* value	*p* value for interaction
**Pooled population**					
Kidney death/failure/40% ↓	404/6316 (6.4%)	451/6321 (7.1%)	0.89 [0.77, 1.01]	0.07	
Kidney death/failure/50% ↓	217/6316 (3.4%)	256/6321 (4.0%)	0.84 [0.70, 1.01]	0.06	
Kidney death/failure/57% ↓	156/6316 (2.5%)	172/6321 (2.7%)	0.90 [0.72, 1.12]	0.34	
**eGFR ≥90 mL/min/1.73m^2**					
Kidney death/failure/40% ↓	110/2112 (5.2%)	116/2156 (5.4%)	0.95 [0.73, 1.24]	0.72	0.37
Kidney death/failure/50% ↓	45/2112 (2.1%)	48/2156 (2.2%)	0.94 [0.63, 1.42]	0.78	0.41
Kidney death/failure/57% ↓	26/2112 (1.2%)	23/2156 (1.1%)	1.14 [0.65, 2.00]	0.65	0.25
**eGFR 60—<90 mL/min/1.73m^2**					
Kidney death/failure/40% ↓	119/2633 (4.5%)	134/2673 (5.0%)	0.90 [0.71, 1.16]	0.42	0.37
Kidney death/failure/50% ↓	54/2633 (2.1%)	62/2673 (2.3%)	0.89 [0.62, 1.28]	0.52	0.41
Kidney death/failure/57% ↓	33/2633 (1.3%)	34/2673 (1.3%)	0.99 [0.61, 1.60]	0.97	0.25
**eGFR 30—<60 mL/min/1.73m^2**					
Kidney death/failure/40% ↓	120/1400 (8.6%)	150/1333 (11.3%)	0.73 [0.57, 0.92]	0.009	0.37
Kidney death/failure/50% ↓	69/1400 (4.9%)	96/1333 (7.2%)	0.65 [0.48, 0.89]	0.007	0.41
Kidney death/failure/57% ↓	50/1400 (3.6%)	71/1333 (5.3%)	0.64 [0.45, 0.92]	0.017	0.25
**eGFR <30 mL/min/1.73m^2**					
Kidney death/failure/40% ↓	55/171 (32.2%)	51/159 (32.1%)	0.98 [0.67, 1.44]	0.92	0.37
Kidney death/failure/50% ↓	49/171 (28.7%)	50/159 (31.4%)	0.89 [0.60, 1.32]	0.57	0.41
Kidney death/failure/57% ↓	47/171 (27.5%)	44/159 (27.7%)	0.97 [0.64, 1.46]	0.89	0.25

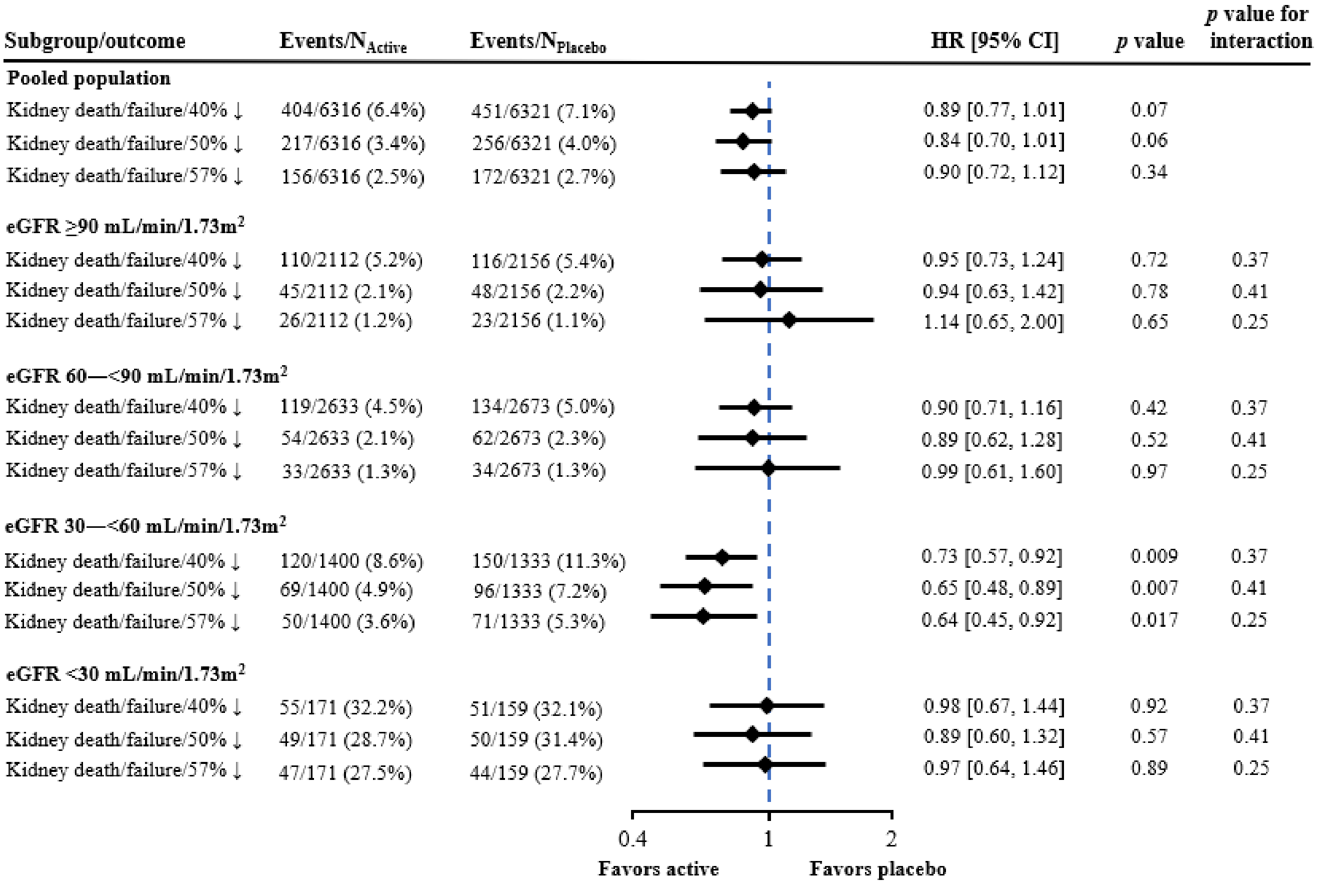

Time to first event analyses were performed using Cox proportional hazard models with pooled treatment as a fixed factor and stratified by trial. Patients without respective events were censored at death or the end of follow-up, whichever came first. Time to persistent reduction of eGFR from baseline (40%, 50% and 57%) was analysed independently from each other. Subgroup analyses were performed by including subgroup as a fixed factor and the interaction between subgroup and treatment. CI, confidence interval; eGFR, estimated glomerular filtration rate; HR, hazard ratio.

Supplementary Figure 6. Effects of semaglutide/liraglutide versus placebo on the risk of composite kidney outcomes (kidney death, kidney failure or proportional eGFR decline [40%, 50% and 57%]), in the pooled population and subgroups defined by the level of proteinuria at baseline.

Subgroup/outcome	Events/N_{Active}	Events/$N_{Placebo}$	HR [95% CI]	*p* value	*p* value for interaction
**Pooled population**					
Kidney death/failure/40% ↓	404/6316 (6.4%)	451/6321 (7.1%)	0.89 [0.77, 1.01]	0.08	
Kidney death/failure/50% ↓	217/6316 (3.4%)	256/6321 (4.0%)	0.84 [0.70, 1.01]	0.06	
Kidney death/failure/57% ↓	156/6316 (2.5%)	172/6321 (2.7%)	0.90 [0.72, 1.12]	>0.15	
**Normoalbuminuria**					
Kidney death/failure/40% ↓	115/3842 (3.0%)	113/3807 (3.0%)	0.99 [0.77, 1.29]	>0.15	0.39
Kidney death/failure/50% ↓	47/3842 (1.2%)	42/3807 (1.1%)	1.09 [0.72, 1.66]	>0.15	0.45
Kidney death/failure/57% ↓	24/3842 (0.6%)	23/3807 (0.6%)	1.02 [0.58, 1.81]	>0.15	0.78
**Microalbuminuria**					
Kidney death/failure/40% ↓	118/1695 (7.0%)	118/1645 (7.2%)	0.97 [0.75, 1.25]	>0.15	0.39
Kidney death/failure/50% ↓	45/1695 (2.7%)	46/1645 (2.8%)	0.95 [0.63, 1.43]	>0.15	0.45
Kidney death/failure/57% ↓	29/1695 (1.7%)	26/1645 (1.6%)	1.09 [0.64, 1.84]	>0.15	0.78
**Macroalbuminuria**					
Kidney death/failure/40% ↓	163/657 (24.8%)	210/729 (28.8%)	0.81 [0.66, 1.00]	0.046	0.39
Kidney death/failure/50% ↓	121/657 (18.4%)	160/729 (21.9%)	0.81 [0.64, 1.03]	0.09	0.45
Kidney death/failure/57% ↓	99/657 (15.1%)	119/729 (16.3%)	0.90 [0.69, 1.17]	>0.15	0.78
**Micro- or macroalbuminuria**					
Kidney death/failure/40% ↓	281/2352 (11.9%)	328/2374 (13.8%)	0.85 [0.72, 0.99]	0.042	0.30
Kidney death/failure/50% ↓	166/2352 (7.1%)	206/2374 (8.7%)	0.80 [0.65, 0.99]	0.036	0.19
Kidney death/failure/57% ↓	128/2352 (5.4%)	145/2374 (6.1%)	0.88 [0.69, 1.12]	>0.15	0.64

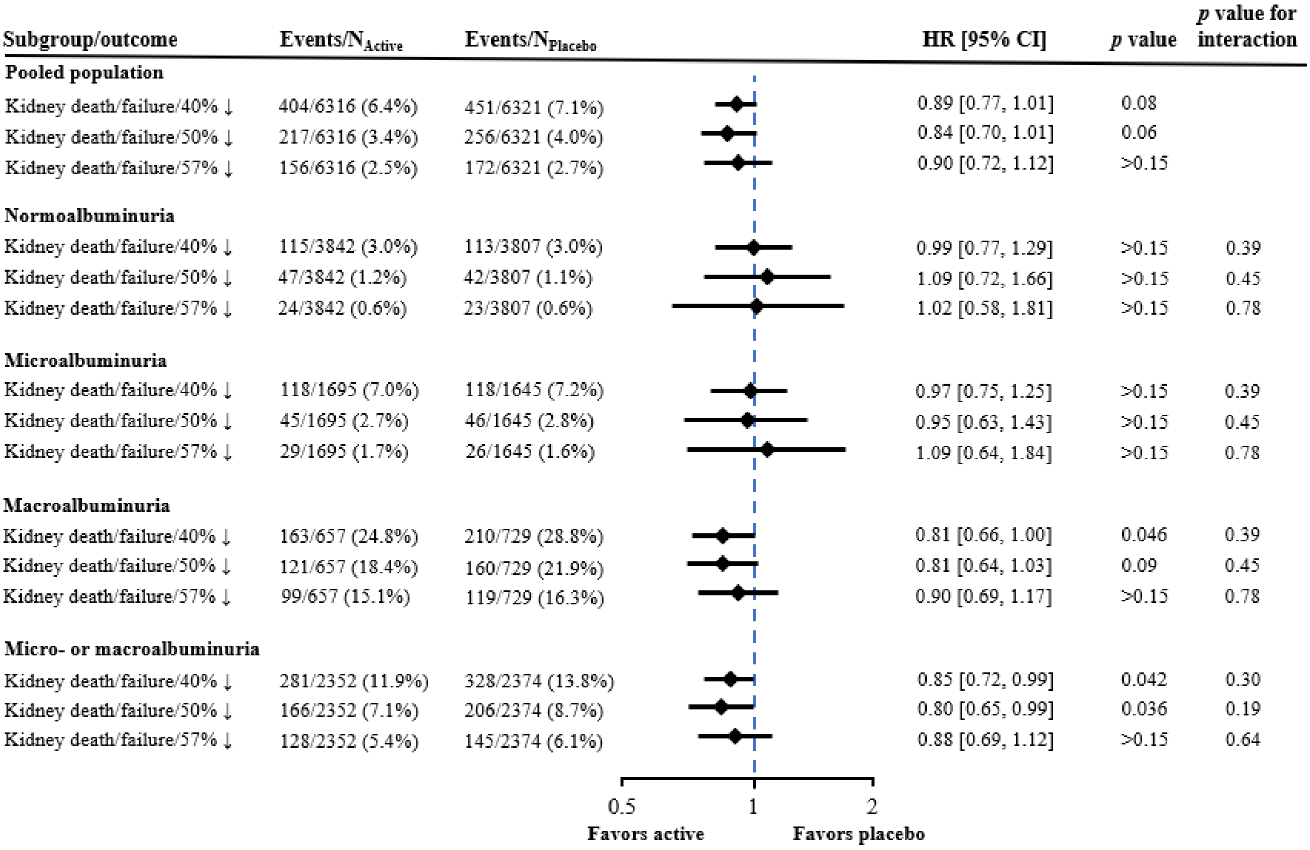

Time to first event analyses were performed using Cox proportional hazard models with pooled treatment as a fixed factor and stratified by trial. Patients without respective events were censored at death or the end of follow-up, whichever came first. Time to persistent reduction of eGFR (40%, 50% and 57%) from baseline was analysed independently from each other. Subgroup analyses were performed by including subgroup as a fixed factor and the interaction between subgroup and treatment. CI, confidence interval; eGFR, estimated glomerular filtration rate; HR, hazard ratio.

Supplementary Figure 7. Effects of semaglutide/liraglutide versus placebo on the risk of composite kidney outcomes (kidney death, kidney failure or proportional eGFR decline [40%, 50% and 57%]) in the pooled population and subgroups with pre-existing CKD defined by eGFR (mL/min/1.73m^2) and the level of proteinuria at baseline.

Subgroup/outcome	Events/N_{Active}	Events/$N_{Placebo}$	HR [95% CI]	*p* value	*p* value for interaction
**Pooled population**					
Kidney death/failure/40% ↓	404/6316 (6.4%)	451/6321 (7.1%)	0.89 [0.77, 1.01]	0.08	
Kidney death/failure/50% ↓	217/6316 (3.4%)	256/6321 (4.0%)	0.84 [0.70, 1.01]	0.06	
Kidney death/failure/57% ↓	156/6316 (2.5%)	172/6321 (2.7%)	0.90 [0.72, 1.12]	0.34	
**eGFR >60 mL/min/1.73m^2 or normoalbuminuria**					
Kidney death/failure/40% ↓	248/5423 (4.6%)	275/5488 (5.0%)	0.91 [0.76, 1.08]	0.26	0.21
Kidney death/failure/50% ↓	104/5423 (1.9%)	122/5488 (2.2%)	0.86 [0.66, 1.11]	0.25	0.50
Kidney death/failure/57% ↓	61/5423 (1.1%)	65/5488 (1.2%)	0.94 [0.67, 1.34]	0.75	0.38
**eGFR 30—60 mL/min/1.73m^2 & micro- or macroalbuminuria**					
Kidney death/failure/40% ↓	100/723 (13.8%)	123/679 (18.1%)	0.70 [0.54, 0.91]	0.008	0.21
Kidney death/failure/50% ↓	63/723 (8.7%)	81/679 (11.9%)	0.68 [0.49, 0.94]	0.021	0.50
Kidney death/failure/57% ↓	47/723 (6.5%)	61/679 (9.0%)	0.68 [0.47, 1.00]	0.049	0.38
**eGFR <30 mL/min/1.73m^2 & micro- or macroalbuminuria**					
Kidney death/failure/40% ↓	53/132 (40.2%)	50/122 (41.0%)	0.98 [0.67, 1.44]	0.92	0.21
Kidney death/failure/50% ↓	47/132 (35.6%)	50/122 (41.0%)	0.87 [0.58, 1.30]	0.50	0.50
Kidney death/failure/57% ↓	45/132 (34.1%)	44/122 (36.1%)	0.95 [0.63, 1.45]	0.82	0.38

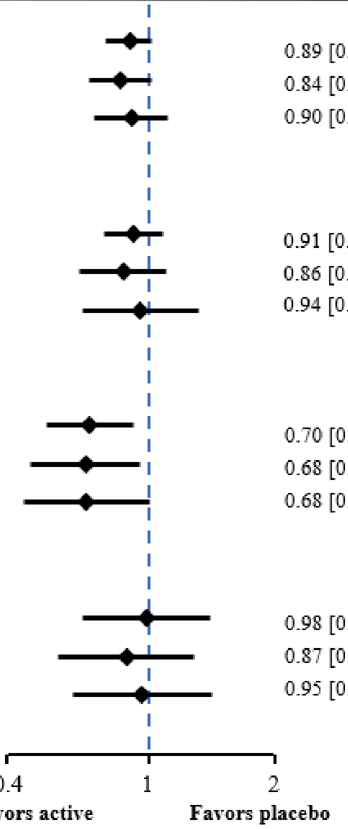

Time to first event analyses were performed using Cox proportional hazard models with pooled treatment as a fixed factor and stratified by trial. Patients without respective events were censored at death or the end of follow-up, whichever came first. Time to persistent reduction of eGFR (40%, 50% and 57%) from baseline was analysed independently from each other. Subgroup analyses were performed by including subgroup as a fixed factor and the interaction between subgroup and treatment. CI, confidence interval; eGFR, estimated glomerular filtration rate; HR, hazard ratio.
